# A Novel Hybrid Feature Extraction Model for Classification on Pulmonary Nodules

**DOI:** 10.31557/APJCP.2019.20.2.457

**Published:** 2019

**Authors:** S Piramu Kailasam, M Mohamed Sathik

**Affiliations:** 1 *Research Scholar, Research and Development Centre, Bharathiar University,Coimbatore, *; 2 *Principal, Sadakathullah Appa College, Tirunelveli,Tamil Nadu, India. *

**Keywords:** Deep learning, convolutional neural network, feature, descriptor, classification

## Abstract

In this paper an improved Computer Aided Design system can offer a second opinion to radiologists on early diagnosis of pulmonary nodules on CT (Computer Tomography) images. A Deep Convolutional Neural Network (DCNN) method is used for feature extraction and hybridize as combination of Convolutional Neural Network (CNN), Histogram of Oriented Gradient (HOG), Extended Histogram of Oriented Gradients (ExHOG) and Local Binary Pattern (LBP). A combination of shape, texture, scaling, rotation, translation features extracted using HOG, LBP and CNN. The Homogeneous descriptors used to extract the feature of lung images from Lung Image Database Consortium (LIDC) are given to classifiers Support Vector Machine (SVM), K-Nearest Neighbour (KNN), Decision Tree and Random Forest to classify nodules and non-nodules. Experimental results demonstrate the effectiveness of the proposed method in terms of accuracy which gives best result than the competing methods.

## Introduction

Lung Cancer is one of the risky malignancies more than breast, colon and prostate cancers combined (Niranjana and Ponnavaikko, 2017). In Japan, Lung cancer is the leading cause of cancer deaths of men 25% in 2015. Globally, Statistical report says 80% cigarette smoking is the root cause. Beginning stage diagnosis could improve survival period. Pulmonary nodules which is spherical or oval sized, origin of cancer. Computerized Tomography (CT) is better in image quality, level of detail and resolution than other modality. Cancerous lung nodule, small mass of tissues , appears round in shape requires treatment. Computer aided diagnosis in clinical procedures can support in making the decision of malignant nodule (Rabia et al., 2017). The Lung Image Database Consortium dataset is used. The pre-processing of the nodule is complicated task. Convolutional Neural Network in Deeplearning preprocessed the lung CT images automatically, leading superior performance. A good feature extraction technique and feature descriptor should be capable of extracting the required features for nodule recognition. Deep learning architecture convolutional neural network is capable of extracting nodule recognition in hierarchical manner by using multiple layers of convolution and maxpooling. Deep Learning has been shown in to extract feature pattern and classify nodules by modifying its architecture and improve the classification time. Deep learning techniques can potentially change the design paradigm of the systems without need of explicit design (Jie-Zhi et al., 2016). The heterogeneous features of CNN as well as feature descriptors score the radiologist ratings (Sihong et al., 2016). Deep Learning Convnet technique is well-matched for false positive reduction of a CAD system (Goharian et al., 2003). The low computation time of ConvNets is a decision aid in lung cancer screening (Apte et al., 1997). The proposed framework, in order to understand the lung nodule features, heterogeneous computational feature type derived from the convolutional neural network (CNN) as well as general low level histogram of oriented gradients (HOG), Extended Histogram of Oriented Gradients (ExHOG) and Local Binary pattern features, exactly detect the nodules in CT pulmonary images. Further more extracted features from hybridized descriptors are classified using excellent classifiers SVM, KNN, Decision Tree and Random Forest methods.

*Related Works*

Pulmonary lung images are to be addressed in four stages, in the order of occurrence namely Deep learning Convolutional Neural Network(DCNN) architecture formation, Image Enhancement, Feature Extraction and Classification.

DCNN is an automated Preprocessing architecture so no need to do preprocessing again. In Deep Learning images or videos may profit from preprocessing , whose job may become much easier (Jodogne and Piater, 2007). A dominant dictionary like texture descriptor, texton is proposed as a feature (Beijbom et al., 2012). Eduardo et al have used a wavelet filters to extract texture feature from open CV library have determined the counts of corals by extracting texture features using LBP descriptor because they use the information from eight directions around a pixel (Huan et al., 2010). CNN were introduced by Lecun et al., (1998), CNN allows for multiple features to be extracted at each hidden layer. Hence, multiple characteristics can be extracted and the learning process assigns weights appropriately to significant features. So automatically performing the difficult task of feature engineering in hierarchically. Shape features (Ashis et al., 2016) can easily extracted by HOG (Histogram of Oriented Gradients)feature descriptor (Chen et al., 2014). The other method to detect whole human detection using (Extended Histogram of Gradients) ExHOG feature descriptor (Amit et al., 2011). Support Vector Machine (SVM) produce good classification for medium size data set. K–Nearest Neighbor(KNN) with Euclidean distance as classifier which is log likelihood and have reported an accuracy of 80%. Upon comparison , that the decision tree yields the most accurate performance and SVM results in poor performance. Decision Tree (Golmohammeadi et al., 2007), Random Forest (Golmohammadi et al., 2007; Hayat and Khan, 2012a) are other classification methods. In this paper various feature descriptors and classifiers are used to extract the lung image nodule and classify with improved accuracy in deep learning environment.

## Materials and Methods

*Data set*

The proposed classification framework and the competing technique deep learning is evaluated over a data set of 467 nodules of Lung Image Database Consortium (LIDC) public database. Each slice of lung CT images consists of 512 X 512 pixels. The pixel resolution starts from 0.5 to 0.8mm. The nodule, noduleid, slice number, diameter are available. The Lung Image Database Consortium (LIDC) dataset is accessible in public for the medical imaging research community (Apte et al., 1997). LIDC dataset contains 1018 Pulmonary CT images that originated from a total of 1010 patients , in total 2,43,958 images. 

*Deep Learning*

Deep learning is cascade of nonlinear transformation in hierarchical mode. Convolution net (ConvNet) feedforward deep architecture end to end learning is applied well. In convolution net each layer can visualize features and have high variance (Tara et al., 2013). At test time, Convnet, run only in forward mode and all layers are differentiable. ConvNet trying to match every possible pieces of image. The convolution layer linearizes manifold and pooling layer collapses manifold. In convolutional layer size of the output is proportional to the number of filters and depends on the stride (Prajwal et al., 2016). Assume filter is an eye detector. If kernels have size C x C, input has size F x F, stride is 1 and there are M input feature maps and N output feature maps then the input has size M@F x F , the output has size N@(F-C+1) x (F-C+1), the kernels have M X N X C X C coefficients which have to be learnt and cost M x C x C x N x (F-C+1) x (F-C+1).The size of the filters has to match the size / scale of the patterns we want to detect. The output size of pooling layer is depends on the stride between the pools. For instance if pools do not overlap and have size C x C and the input has size F x F with M input feature maps then the output is M @ (F / C) x (F / C) (Qingzheng et al., 2017).

 f = f ɭ o f ɭ -1 o ..........f1                     (1)

Each function f ɭ represents a layer which takes the output of the previous layer , denoted by x ɭ -1 to compute the output x ɭ using the parameters w ɭ equipped for each layer, 

ie. x_ɭ _= f_ɭ_ (x_ɭ -1_; w_ɭ_ ) for ɭ = 2,3,....L

*Feature Extraction*

Shape based features are computed from the biggest axial slice of nodule (Rangayan et al.,1997).The shape based low level features are Area, Perimeter, Compactness, Major axis length, Minor axis length, Equilent diameter, Convex area, Convex perimeter and Circularity. Low level feature which is shape based is calculated by HOG, ExHOG feature descriptor and texture feature calculated by LBP feature descriptor. High level features translation , scaling and rotation are calculated by Convolution Neural Network Convnet. When both descriptors are hybridized to get low and high level feature extraction, the resultant image shows excellent performance which matches with train nodule features. 

*Blocks of Convolutional Neural Network as Feature Descriptor*

In this paper on the performance of the 2D CNN with LIDC dataset, the first layer of the CNN is a convolutional layer with filter size 20, stride size of 1, followed by a max pooling layer of size 2 x 2 with stride size of 1. The third layer is also a convolutional layer with filter size 32 and the stride size as layer 1. The first six layers are arranged alternately by convolution layer1, max pooling layer1, convolution layer 2, max pooling layer 2, convolution layer3, max pooling3 pattern, except that the fifth layer is with filter of size 32. The seventh layer is an activation layer with ReLU (Rectified Linear Unit) and the eighth layer is again a convolutional layer with filter size 40 @ 4 x 4 x 32. The last layer is a softmax operator. The parameters of filters in the convolutional layers and the size of max pooling operators must be consistent to allow meaningful computations. For our datasets, each input image of size 50 x 50 x 1 leads to an output of size 1 x 1 x 2 after forward propagation of the 9 layers (Yu et al., 2016; Qingzeng et al., 2017). 

CNN consists of convolutional layers which are characterized by input Image I, bank of filters K of dimension k1 X k2 with height ‘h’ ,weight ‘w’ and biases ‘b’. The output from this convolution procedure is as below

${\left(\mathrm{I*K}\right)}_{x,y}=\sum_{i=1}^{h}\sum_{j=1}^{w}{\mathrm{kji.I}}_{X+i-1,y+j-1}+b$

 (2)

*HOG (Histogram of Oriented Gradients) Feature Descriptor*

The image resized into 28 x 28 is decomposed into 6 x 6 overlapping blocks where each block is 2 x 2 cell with stride size of 4 pixels. The number of bins are 9 .There are totally 1,296 HOG features computed as lowlevel shape features. To improve accuracy the local histograms can be contrast normalized. In block larger region of image calculates the intensity measurement. The pulmonary image get good intensity and shadowing in normalization. This operated on local cells. Here opposite directions are put in to same bin and calculated as same orientation. The angle range is 0 to 180 degree. To filter color or intensity of the image gradient computation in horizontal and vertical directions , (-1 0 1) and (-1 0 1)T are necessary (Amit saptapathy,2011).

The gradient magnitude(M) of each pixel (x,y) and its direction , theta are computed as follows:

$M\left(x,y\right)=\sqrt[{2}]{{\mathrm{Ix}}^{2}}+{\mathrm{Iy}}^{2}$

 (3)

M(x,y) is the magnitude of pixel.

We can calculate pixel direction (x,y) , 

$\theta =\tan^{-1}\frac{\mathrm{ly}}{\mathrm{lx}}$

 (4)

here the theta intervals between o to 2π. Where Ix and Iy are gradients in the x and y directions.

*ExHOG (Extended HOG) Feature Descriptor*

ExHOG is a feature descriptor which extract HOG features around corner points. First read the nodule image and divided into two halfs as A and B. Through Histogram Image, the values A+B and A-B are calculated. Detect the strongest corners in the image and difference of (A+B) and (A-B) is the EXHOG feature descriptor. (I, Strongest) is equal to (ExHOG, Validpoints, p).The bin value is 18 and window is 6 x 6. So in total 648 bins formed. Extended Histogram of Gradient (ExHOG) is a excellent feature descriptor which concatenate Difference of HOG and HOG of L/2 bins each to form a histogram of L bins for each cell in the image window(Amit,2011). Then the feature block is concatenated and normalized by clipping method.

The equation summarizing the steps is as follows:


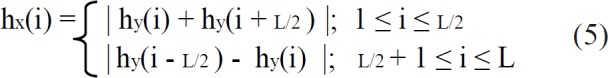


where hx (i) is the ith bin value of ExHoG, hy(i) is the ith bin value of HG and L is the number of bins in HG and ExHoG, correspondingly. The features from all the blocks are collected for the image window to form a combined feature vector.

*Local Binary Pattern*

Local Binary Pattern (LBP) is an efficient technique for texture feature extraction. It considers that each pixel is compared with neighbour pixels and encodes the binary effect of the local neighbours by simple threshold function (Ani Brown Mary et al., 2017). The gray value of the neighbouring pixels are higher or equal to the centre pixel, the value is set to one or set to zero. If k is the total number of neighbouring pixels , then LBP technique feature results in 2k dimensions of feature vector. This makes 256 patterns for eight neighbour pixels and 65,536 patterns for 16 neighbour pixels. As the size of the neighbourhood increases, the size of feature vector also increases. The histogram of binary of pulmonary image, patterns obtained for a region is considered as feature descriptor (Armato et al., 2000). 


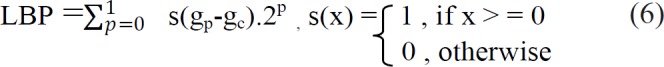


where g_p_ is neighbour pixel, g_c_ central pixel

*Classification*

Support Vector Machine is supervised learning model that is trained with nodule and nonnodule CT images by means of extracted feature values. Both classes are divided by a hyperplane, which derives a kernel to separate the classes. Also SVM is memory efficient classifier. K Nearest Neighbour Classifier(K-NN) is one of the simplest classifiers that identifies the undefined data by previously known data and classify them by voting system. K-NN classifies the nodule points using more than one nearest neighbour (Wang et al., 2005; 2008; 2010a; 201b). In KNN classification , the output is a class membership, if k = 1 then the object is simply assigned to the class of nearest neighbour.

Decision Tree predict responses to data in a tree structure. Each node denotes a test on an element value. Each branch represents an outcome of the test. This follow decisions in the tree from the parent node down to a child node. The child node contains the response. Decision tree responses that are nominal, such as ‘1’ or ‘2’. The tree predicts classifications based on two predictors which is 1 or 2 in feature extracted nodule images (Qi et al., 2016). The approach of this tree is widely used since it is efficient, can deal with both continuous and categorical variables and generates understandable rules (Apete, 1997). Also it is able to deal with missing values in the training data and can also tolerate some errors in the data. Furthermore , decision trees can lead to large errors if the number of training examples per class is small. Random Forest (RF) is a collection of decorrelated decision trees and good in large data set. RF and regression trees are an ensemble of classification. (CART) (Heet al., 2016). The error rate of the classification of all the test sets is the out of bag estimate of the generalization error (Alif Hayat, 2015).

*Psuedo Code of Proposed method*

// Let us Consider CT slice as i and Given nodule region (i-2,i-1,i,i+1,i+2). Train nodules (1...n) from 3DCT image of LIDC . Extract feature of nodule using LBP, HOG, CNN, EXHOG//

Begin

d(s)← 1...n

While (d(s) =! eof)

{

HOGfea←HOG(nod_reg(i-2,i-1,i,i+1,i+2))

EXHOGfea ← EXHOG(nod_reg(i-2,i-1,i,i+1,i+2))

CNNfea←2DCNN(fL o f L-1 o…….f1)

LBPfea←LBP(nod_reg(i-2,i-1,i,i+1,i+2))

// Store features of nodules in FV //

FV←(LBPfea HOGfea CNNfea EXHOGfea)

FV1←(CNN_fea_+LBP_fea_ CNN_fea_+HOG_fea _CNN_fea_+EXHOG_fea_......)

CL←AssignClassLabel(noduleregion_1....n_)

// Test the data and use kfold validation as 5 and classify using SVM Classifier //

Classify←(SVMClassify(FV1,CL,testnodreg),RF(FV1,CL,testnodreg),DT(FV1,CL,testnodreg),KNN(FV1,CL,testnodreg))

for j ← 1 to n

if Classify((j)) ← 1

print nodule

else 

print nonnodule

endfor

}

End

## Results

In this study convolutional neural network is made in deeplearning framework and accuracy was calculated. A total of 467 images of lung nodules and nonnodules 131 used in the training. Training set learnt properties are applied to tested data. A five fold validation is used in the combination of {(10,90) (20,80) (30,70) (40,60) (50,50)} where each set denotes (train, test) data. Total pulmonary images are tested in CNN and the same is applied for HOG, EXHOG and LBP descriptors.

The total execution time of hybridized techniques LBP, HOG, ExHOG, CNN with classifiers SVM, KNN, DT, RF are tabulated as shown in Table.1 to 4 seperately. The interative variables are incrementing as per iterations . The time value of SVM_ExHOG_CNN is 5.332s. This is higher than KNN_LBP execution time 2.233s which is least value.

Tables 5 to 8 displays the result obtained. A sample image from each combination is used to create the test set, while remaining samples construct the training set. Totally 598 samples are used for training and 420 for testing. The similar measures are calculated. From this matrix the number of instances that are accurately and inaccurately predicted is easily known. From Table.1, SVM with HOG, HOG_CNN and CNN_LBP descriptors got least False Positive value as 14. The proposed best hybrized feature descriptor EXHOG_CNN with SVM classifier is highlighted and the corresponding TP, TN, FP, FN values are 452, 118, 15, 13 correspondingly. Similarly the other confusion matrix results are also displayed above.

3.3 Comparing different combinations of methods

The highest precision value is 97.23% for SVM_ExHOG_CNN. The highest recall value among 36 methods is 99.78% for KNN_HOG method.

*Convolution Neural Network*

In the deep network during training stage convolution layers consist of 20 filters. The kernel size is 5. The pooling layer has a kernel size of 2. Convolution layers and pooling layers are arranged alternatively three times. ReLU layer is activation layer. Softmax layer which is output layer extracted the feature of nodule as shown in Figure 19. The average of accuracy is 84.59% with maximum iteration 1770 in CNN feature extraction. 

*Hybridized feature descriptors*

HOG, ExHOG, LBP feature descriptors have low level features which is shape or texture. High level feature scaling , translation, shearing and rotation is taken from CNN. By combining the low level and high level features we get the shape features of the nodule which is hetrogenous feature compution(Sihong Chen, Jing Qin et al,2016). The hybridized feature descriptors output of CNN+HOG, CNN+EXHOG, CNN+LBP are taken and then classified by SVM , KNN, Random Forest, Decision Tree classifiers. 

The Combination CNN and LBP formula,


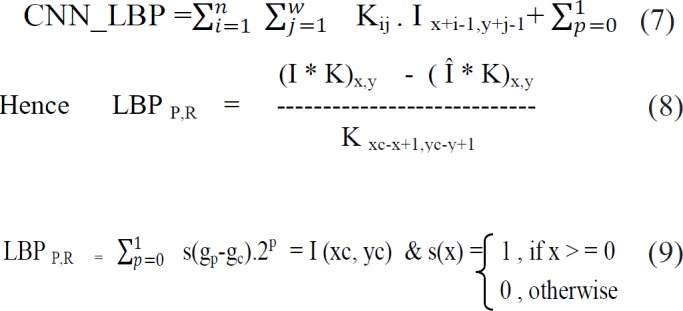


The hybridized CNN and HOG became as,


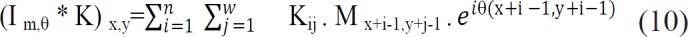


Next HOG and LBP mathematically expressed as,


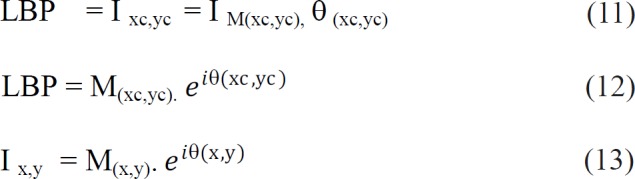


where LBP of an image can be converted in to magnitude ‘M’ and angle ‘θ’.

*Analysis of classification with different combination of feature descriptors*

The Classifiers SVM, K-NN, Decision Tree and Random Forest are used for the classification of nodules and nonnodules. The details of patientid, nodule, noduleid, slicenumber, zposition and diameter are the data collected from LIDC dataset. Here TN and TP values are classified by hyperplane distance in SVM Classifier. All data are tested and trained. Seperate training and test data sets are constructed using k-fold cross validation approach, the value of k taken as five. In k fold validation 467 nodules and 131 nonnodules are validated and implemented in matlab 2017a. The small round or oval shaped growth in lung size less than or equal to 3mm in diameter is nodule. In the Region of Interest (ROI), the nodule <=3mm assigns the value one or else the value is zero. The nodule intensity is 21 x 21. In CT input dicom images, the nodule region of each five slices taken as p2,p1,n,n1,n2 and trained well. Here ‘n’ is Region of interest. The Positive Predictive Value (PPV) and Negative Predictive Value (NPV) are calculated from confusion matrix. The PPV value of SVM_LBP is 92%. The PPV value of SVM_CNN is 91.8%, lesser than SVM_LBP. The PPV value of SVM_HOG_CNN, SVM_ExHOG and SVM_HOG_CNN_LBP are 97% ,96.5% and 93% respectively. The NPV value of SVM_LBP is 79.3%. The SVM_CNN, SVM_LBP, SVM_ExHOG, SVM_HOG_CNN_LBP have NPV values as 65.64%, 79.3%, 80.15%, 81.6% vice versa.

The output of hybridized feature descriptors CNN, LBP, HOG, ExHOG combined in various patterns and SVM classifier classified the nodules as shown in Table1 and 2. The results depict that the proposed method performed well. The TP, TN, FP, FN values are taken from confusion matrix Table1 and Table2. The performance of SVM classifier is calculated using various metrics such as Accuracy, Sensitivity, Specificity, Cohen’s Kappa, Fscore, Precision and Recall. The accuracy of classification based on SVM classifier for both training and test data was evaluated by chi square test at confidence level. The highest accuracy value of SVM_ExHOG_CNN is 95.32% as result. The performances of the SVM Classifier is compared with KNN, Decision Tree and Random Forest Classifier. The KNN classifier classifies the nodule and nonnodule with K value one and the accuracy 94.15% is obtained. A decision tree is a non-parametric supervised learning method generally used in data mining. The goal is to create a model that predicts the value of target value using decision rules and pruning which inferred from data features. Decision Tree does not work well for simple classification. Random forest uses a number of decision trees in order to improve the classification rate of pulmonary nodule images , depends on the values of a random vector for all trees in the forest. K-NN performs medium level in classification of two classes.

*Time*

Among the determined KNN classifier techniques minimum time of KNN_LBP is 2.333s which is tabulated and the minimum value is bolded. The DT_LBP gives the time value as 2.37s which is lower than SVM classifier. The RF_LBP gives the time value 11.999s which is higher than the other classifiers.

*Accuracy*

Over all Accuracy value is calculated in the combination of SVM_LBP, SVM_HOG, SVM_ExHOG, SVM_CNN, SVM_ExHOG_CNN, SVM_CNN_LBP, SVM_HOG_CNN. The result shows the accuracy value of both SVM_HOG_CNN and SVM_CNN_LBP are the same as 95.15% and the highest accuracy value is SVM_ExHOG_CNN as 95.32%. The value of SVM_ExHOG_CNN value is greater than SVM_HOG, SVM_ExHOG, SVM_CNN, SVM_CNN_LBP, SVM_HOG_CNN combinations. It is noted that SVM classifier is improved in CNN + ExHOG than CNN+HOG . The accuracy value of KNN classifier is good with HOG descriptor as 94.15%. Random forest classifier‘s best accuracy value with HOG is 94.65% and other combinations are greater than 90.64%. Decision Tree classifier with ExHOG combination gives best accuracy value as 88.3%.

$\mathrm{Accuracy}=\frac{\mathrm{TP}+\mathrm{TN}}{\mathrm{TP}+\mathrm{FN}+\mathrm{FP}+\mathrm{TN}}$

 (14)

Where TP is true positive that counts classified foreground pixels. FP counts the background pixels incorrectly classified as foreground. FN false negative that counts foreground pixels incorrectly classified as background. TN counts correctly classified background pixels.

*Specificity*

It is the ability of the classifier to predict the true negatives which is given by the equation

 (15) $$\mathrm{Accuracy}=\frac{\mathrm{TN}}{\mathrm{FP}+\mathrm{TN}}$$

Specificity of the SVM classifier is good since the true negative values are mostly identified. The Specificity value of SVM_ExHOG_CNN combination is 95.03% which is higher than other classifiers KNN, Decison Tree and Random Forest combinations. The value of RF_EXHOG is 57.95% due to less misclassification rate. 

In specificity the maximum value among the results is taken. The Specificity values of KNN, Decision Tree and Random Forest are 81.77% , 80.17, 79.52 % vice versa. 

*Sensitivity*

Sensitivity of both RF_ExHOG and KNN_HOG has value 99.78% and it is higher than other classifiers. 

$\mathrm{Accuracy}=\frac{\mathrm{TP}}{\mathrm{TP}+\mathrm{FN}}$

(16)

*Cohen’s Kappa*

The value of Cohen’s Kappa of SVM_HOG_CNN and SVM_CNN_LBP got same value as 85.78% and the value of RF_HOG is 82.9%.

Kappa Coefficient K=na-nen-ne

(17)

where n_a_ is number of agreements, n_e_ is number of agreements due to chance and n is number of samples.

*F score*

F score is a benchmark metric, which measures image classification accuracy by considering both the recall and precision. Best value of F score is one, while worst is zero. 


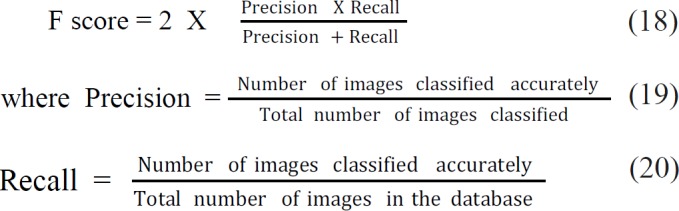


This is a measure that has relation with sensitivity. F score value of SVM_EXHOG_CNN classifier is 97%. 

F score is a measure relative to sensitivity. Generally F score is less for classes with fewer samples and high for classes with larger samples. The F score value of both SVM_CNN_LBP and SVM_HOG_CNN is equal as 96.9%. The Random forest classifier scores the highest value 96.88%. The least value got SVM _CNN and the value is 65.78% .

**Figure 1:**
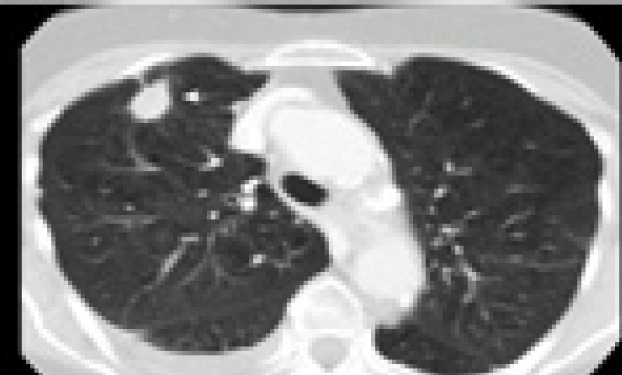
Nodule ≥ 3mm

**Figure 2:**
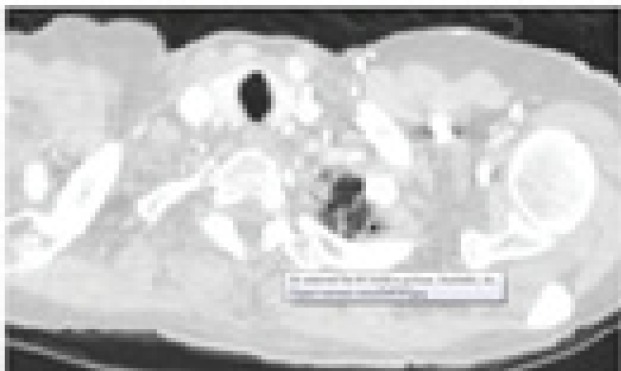
Non Nodule ≥ 3mm

**Figure 3:**
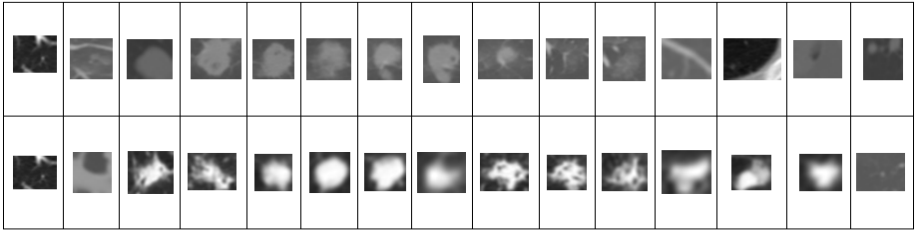
Sample Trained Data

**Figure 4:**
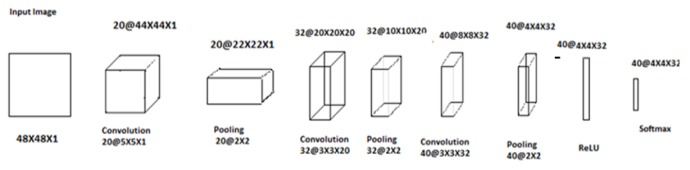
CNN Architecture from the Input Image of Size 48X48

**Figure 5:**
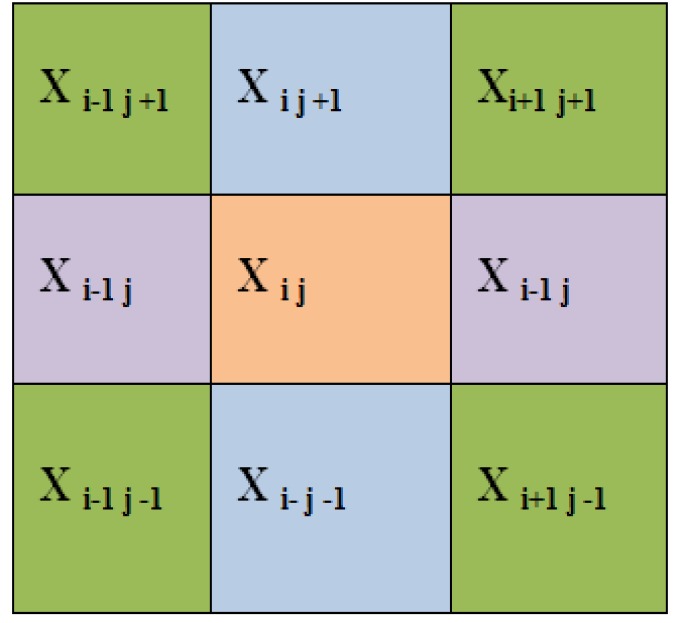
Four Diagonal Pairs of Neighbours of Centre Pixel X_ij_

**Figure 6:**
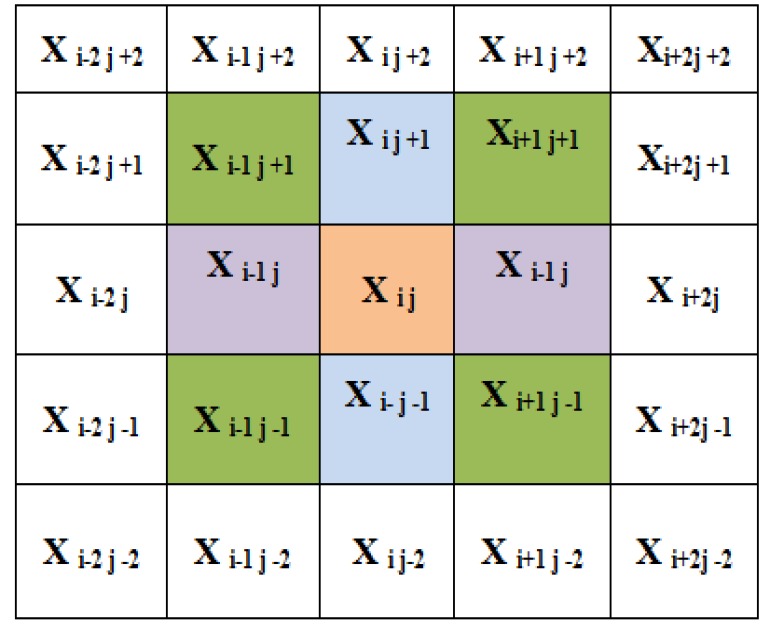
Block Bk of Matrix 5X5 with the Pixel X_ij _and Its Neighbours

**Figure 7:**
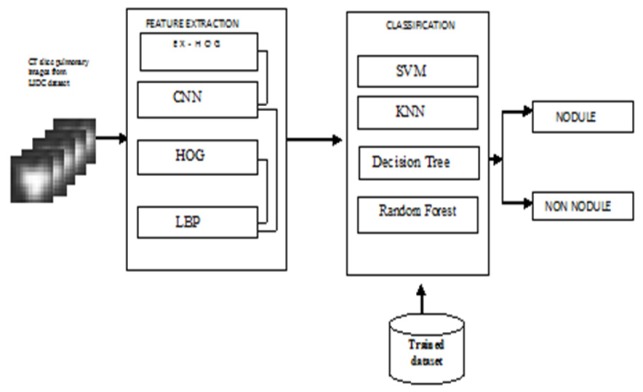
Multi Classifiers with Hybrid Descriptors

**Figure 8:**
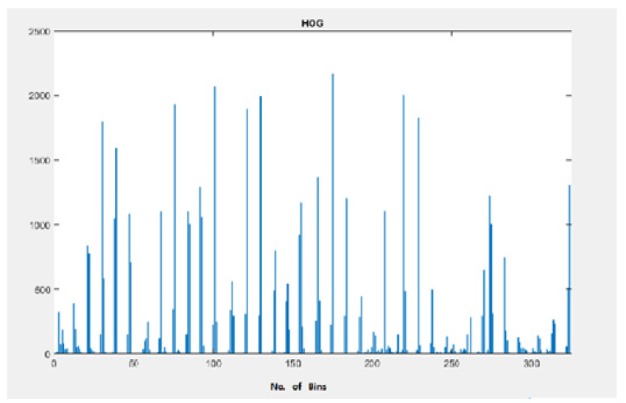
HOG - Histogram of Nodule Image

**Figure 9:**
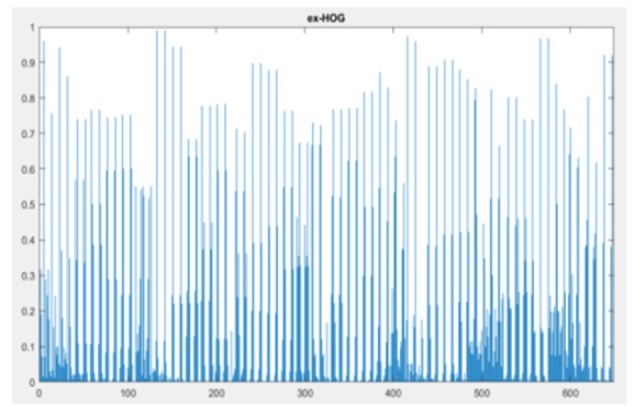
ExHOG-Histogram of Nodule Image

**Figure 10:**
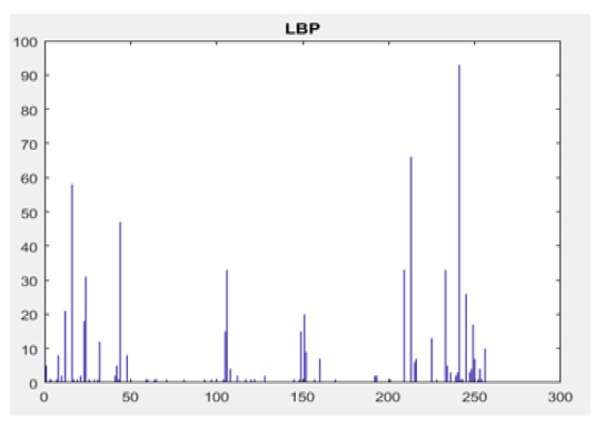
LBP-Histogram of Trained Nodule

**Figure 11:**
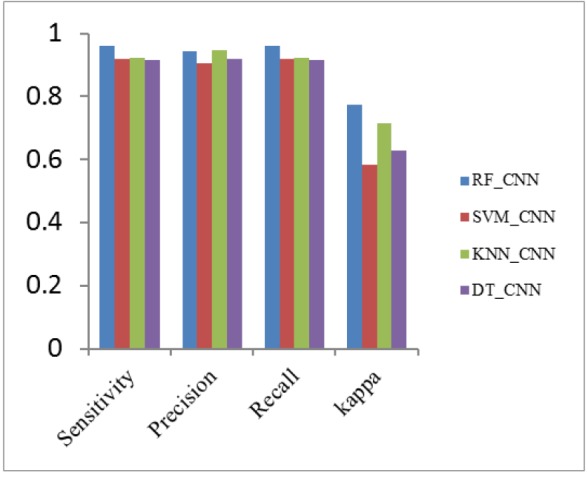
Performance Metrics Analysis of CNN with Classifiers

**Figure 12:**
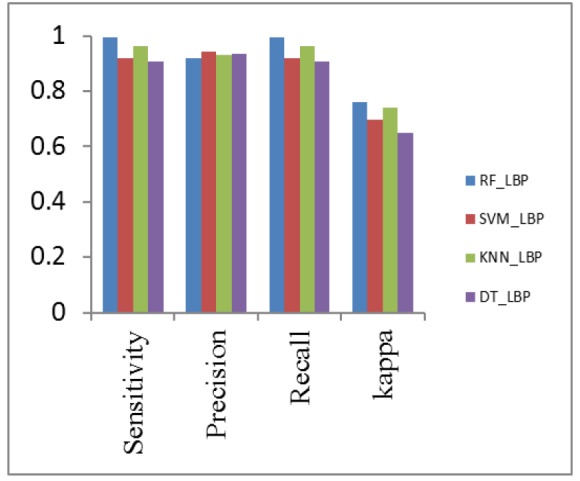
Performance Metrics Analysis of LBP with Classifiers

**Figure 13:**
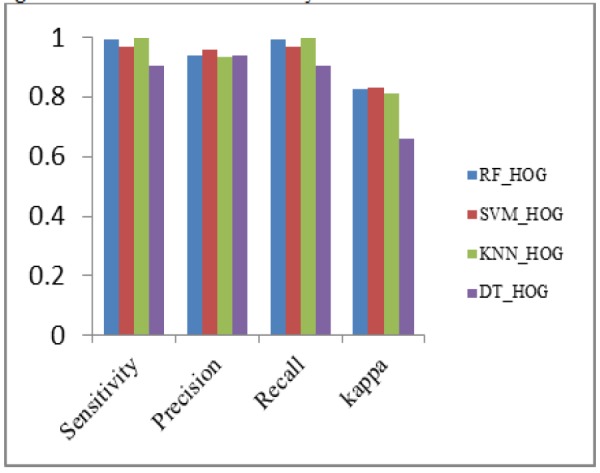
Performance Metrics Analysis of HOG with Classifiers

**Figure 14:**
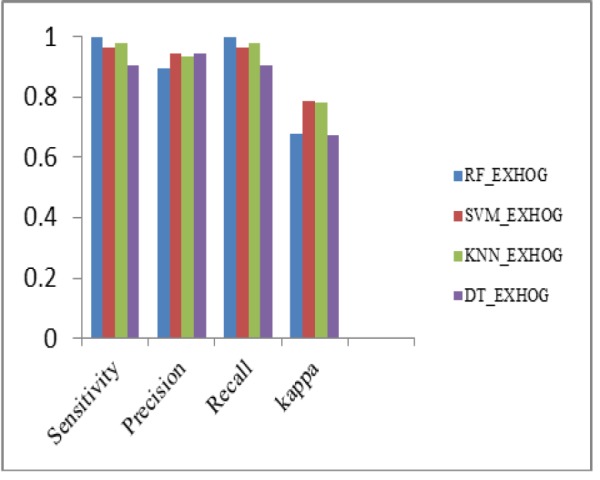
Performance Metrics Analysis of ExHOG With Classifiers

**Figure 15:**
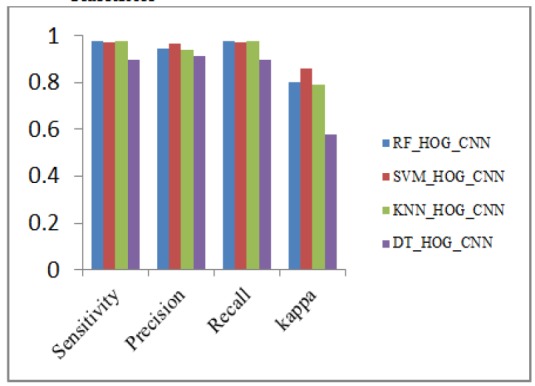
Performance metrics analysis of HOG_CNN with Classifiers

**Figure 16:**
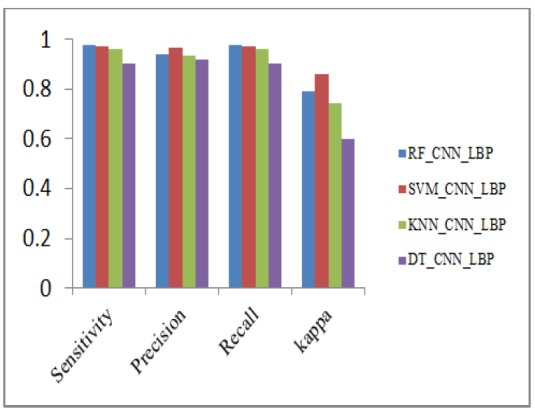
Performance Metrics Analysis of CNN_LBP with Classifiers

**Figure 17:**
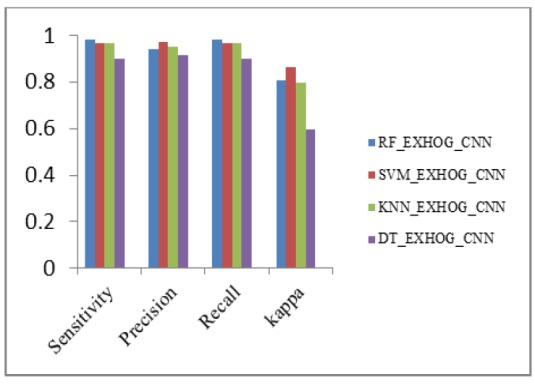
Performance Metrics Analysis of ExHOG_CNN with Classifiers

**Figure18:**
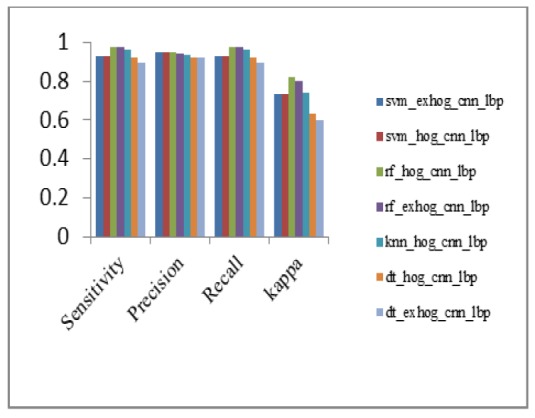
Performance Metrics Analysis of EXHOG_CNN_LBP and with Classifiers

**Figure 19:**
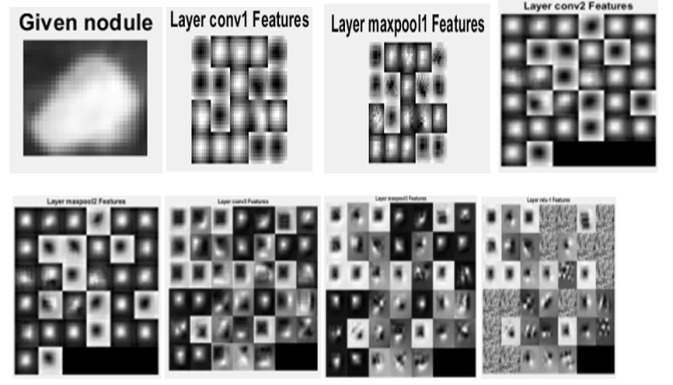
The Output Layers of CNN for Given Nnodule

**Figure 20:**
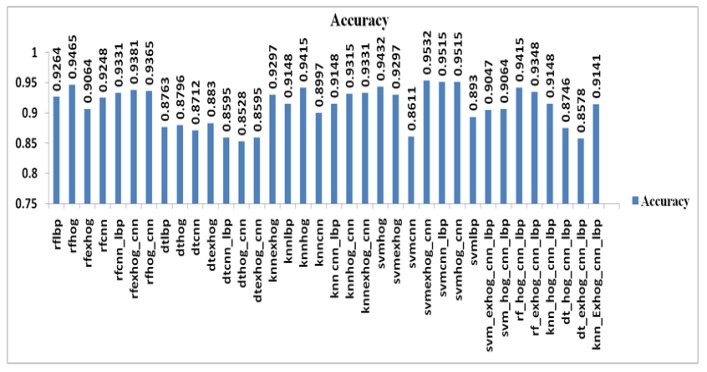
Overall Acuracy

**Figure 21:**
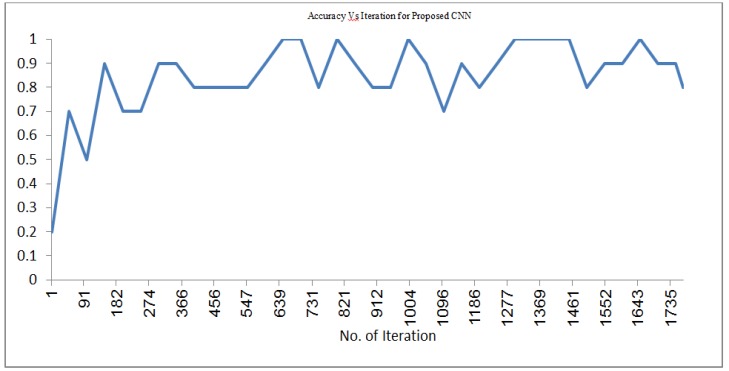
Accuracy Vs Iteration for Proposed Convolutional Neural Network (ConvNet)

**Figure 22:**
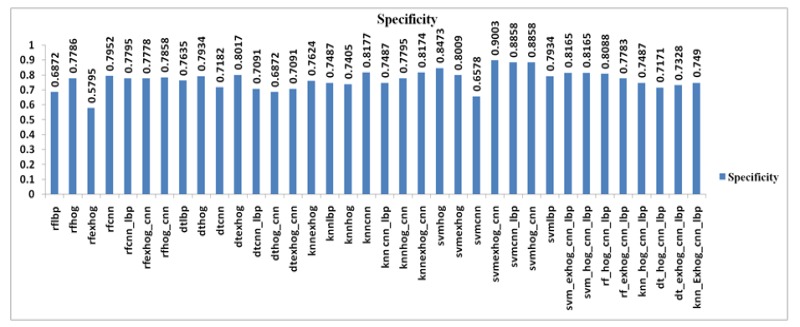
Comparison of Specificity

**Figure 23:**
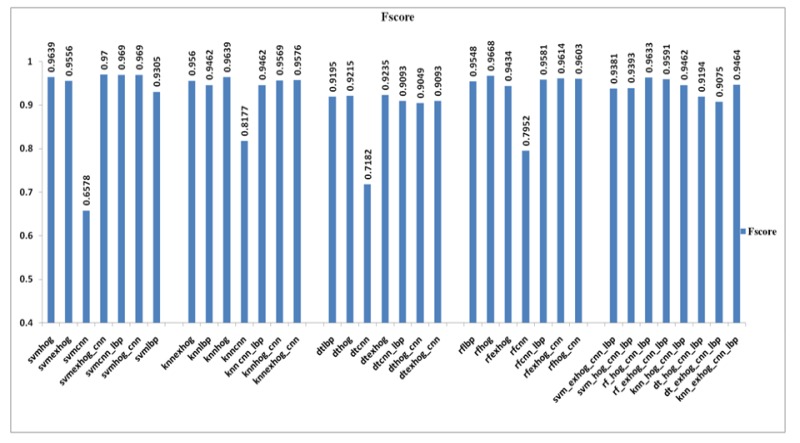
Comparison of F Score

**Table 1:** Time Taken by SVM Classifier Using LBP, HOG, ExHOG, CNN

SVM_LBP	4.265s
SVM_HOG	4.199s
SVM_ExHOG	5.936s
SVM_CNN	3.969s
SVM_HOG_CNN	4.111s
SVM_ExHOG_CNN	5.332s
SVM_CNN_LBP	4.059s
SVM_ExHOG_CNN_LBP	5.908s
SVM_HOG_CNN_LBP	12.386s

**Table 2:** Time Taken by KNN Classifier Using LBP, HOG, EXHOG, CNN

KNN_LBP	2.233s
KNN_HOG	3.414s
KNN_EXHOG	4.902s
KNN_CNN	4.006s
KNN_HOG_CNN	7.518s
KNN_EXHOG_CNN	4.994s
KNN_CNN_LBP	2.387s
KNN_EXHOG_CNN_LBP	5.296s
KNN_HOG_CNN_LBP	3.837s

**Table 3:** Time Taken by RF Classifier Using LBP, HOG, EXHOG, CNN

KNN_LBP	2.233s
KNN_HOG	3.414s
KNN_EXHOG	4.902s
KNN_CNN	4.006s
KNN_HOG_CNN	7.518s
KNN_EXHOG_CNN	4.994s
KNN_CNN_LBP	2.387s
KNN_EXHOG_CNN_LBP	5.296s
KNN_HOG_CNN_LBP	3.837s

**Table 4:** Time Taken by DT Classifier Using LBP, EXHOG, CNN

DT_LBP	2.372s
DT_HOG	4.234s
DT_ExHOG	7.229s
DT_CNN	4.185s
DT_HOG_CNN	4.579s
DT_ExHOG_CNN	7.218s
DT_CNN_LBP	7.390s
DT_ExHOG_CNN_LBP	7.725s
DT_HOG_CNN_LBP	4.923s

**Table 5:** Confusion Matrix of SVM Classifier with Other Feature Descriptors

SVM_ LBP		SVM_ CNN		SVM_EXHOG		SVM_HOG		SVM_HOG_ CNN		SVM_HOG_ CNN_LBP		SVM_EXHOG_ CNN_LBP		SVM_CNN_LBP		SVM_ EXHOG_ CNN	
430	37	429	38	451	16	453	14	453	14	435	32	434	33	453	14	452	15
27	104	45	86	26	105	20	111	15	116	24	107	24	107	15	116	13	118

**Table 6:** Confusion Matrix of Random Forest Classifier with Other Feature Descriptors

RF_LBP		RF_CNN		RF_EXHOG		RF_HOG		RF_HOG_ CNN		RF_HOG_ CNN_LBP		RF_EXHOG _CNN_LBP		RF_CNN_LBP		RF_ EXHOG_ CNN	
464	3	448	19	466	1	464	3	456	11	435	32	457	10	453	14	456	11
41	90	23	108	55	76	29	102	28	103	24	107	29	102	20	111	33	98

**Table 7:** Confusion Matrix of Decision Tree Classifier with Other Feature Descriptors

DT_LBP		DT_CNN		DT_EXHOG		DT_HOG		DT_HOG_ CNN		DT_HOG_CNN_LBP		DT_EXHOG_CNN_LBP		DT_CNN_LBP		DT_ EXHOG_ CNN	
424	43	422	45	423	44	422	45	424	43	429	38	417	50	418	49	418	49
31	100	33	98	26	105	27	104	34	97	37	94	35	96	35	96	35	96

**Table 8:** Confusion Matrix of KNN Classifier with Other Feature Descriptors

KNN_ LBP		KNN _CNN		KNN _ EXHOG		KNN _HOG		KNN _HOG_ CNN		KNN _HOG_CNN_LBP		KNN _EXHOG_CNN_LBP		KNN _CNN_LBP		KNN _ EXHOG_ CNN	
449	18	431	36	451	16	466	1	445	12	449	18	449	18	443	24	451	16
33	98	24	107	24	107	34	97	29	102	33	98	33	98	26	105	24	107

## Discussion

Many researchers used LIDC-IDRI dataset to Classify nodules. A Combination of shape, margin and texture based features for classification pulmonary nodules are segmented using a semi automated technique and classified using SVM classifier to attain accuracy. But Ashis Kumar et al., (2016) is not considered maximum correlation coefficient and provide only seed point for segmentation of nodules. Prajwal et al., (2016) dealed ConNet architecture. ConNet a type of CNN have special properties such as special invariance and allow multiple feature extraction accuracy improvement. ConNet are much better than ANN architecture in accuracy. Qing Zeng et al., (2017) results show that the CNN network achieved the best accuracy of 84.15%. This was the best result among the CNN, DNN and SAE networks. Huan et al., (2010) extracted 14 GLCM textural features to analyze the CT images of benign and malignant pulmonary nodules. The author suggested that need additional variable for explaining texture features in CT images of benign and malignant. He used multilevel binomial logistic prediction model of pulmonary feature of CT images. Goncalves et al., (2016) approaches 3D lung nodule segmentation with Hessian based matrix. He proposed a method validated with 569 solid nodules, provided accurate pulmonary nodule volumes for posterior characters. Shape index and Curvedness approaches are calculated in justavascular nodules. Aranaud et al., (2016) detected multiview convolutional neural network automatically learnt discriminative features from the LIDC data with sensitivities 85.4% at 1 false positive per scan with low computation time to detect nodules. Multitask learning to leverage heterogenous computational features derived from deep learning models of Stacked Denoising Autoencoder (SDAE) and CNN, as well as Hand crafted Haar like and HOG features for the description of a semantic features of lung nodule. The gap between the clinical remark concepts and the low level image features are the major factors that thwarts the retrieval accuracy. The step of computational mapping between the clinical remark terms and image contents to define the similarity measures at semantic level can helped to improve the CBIR performance. In this method the retrieval is based on overall similarity or each specific semantic feature.

In the proposed work, it is observed that the tree based classifiers Decision tree and Random Forest with hybrid feature vectors in deep learning produces lower classification rate ie. approximately 86 – 94 % compared to other classifiers SVM and KNN. Yet SVM can handle the classification of nodules in images well in manner with accuracy rate 95.32%. KNN is also having accuracy as 94.15%. It is noticed that SVM (RBF kernel) can be appropriate classifier for large datasets.

In conclusion, in Deep learning environment, the proposed hybridized feature descriptor is found to be the best for LIDC pulmonary image dataset. This paper presents the test results of classification of the pulmonary image dataset. The feature descriptors CNN, LBP, HOG, ExHOG are hybridized in two or three combinations with various single classifiers that is like SVM, KNN, DT and RF. It may be noted that the SVM_CNN_LBP and SVM_HOG_CNN have classification accuracy which is comparable to the accuracy value of SVM_EXHOG_CNN and the accuracy is 95.15%. SVM_EXHOG_CNN assists in achieving the highest classification accuracy of 95.32% and the execution time is 3s more than KNN_LBP’s execution time 2.233s. Therefore SVM_EXHOG_CNN is 10.04% higher than DT_HOG_CNN in terms of accuracy. Among 36 combinations tried the execution time of KNN_LBP got to be the least with value 2.233s. Considering all the above points, SVM classifier is better than KNN, DT and RF classifiers for the classification of two classes due to its higher accuracy. In future best detection of malignant lung nodule can be achieved by training 2DCNN to 3DCNN with other datasets.
